# The Potential Health Benefits of Polyphenol-Rich Extracts from* Cichorium intybus* L. Studied on Caco-2 Cells Model

**DOI:** 10.1155/2016/1594616

**Published:** 2015-12-29

**Authors:** Elena Azzini, Giuseppe Maiani, Ivana Garaguso, Angela Polito, Maria S. Foddai, Eugenia Venneria, Alessandra Durazzo, Federica Intorre, Lara Palomba, Maria L. Rauseo, Ginevra Lombardi-Boccia, Fabio Nobili

**Affiliations:** Council for Agricultural Research and Economics (CREA), Research Center for Food and Nutrition, Via Ardeatina 546, 00178 Rome, Italy

## Abstract

Phytochemicals can exert their bioactivity without reaching the systemic circulation; scarcely absorbed antioxidants might reach the large bowel contributing to protection from oxidative damage-induced gastrointestinal diseases. In the present work, we aimed to study the relationship between potential activity of polyphenol-rich extracts from *Cichorium intybus* L. and changes in morphological characteristics on Caco-2 cells. Phytochemicals content (carotenoids and flavonoids) and total antioxidant activity of Red Chicory of Treviso and Variegated Chicory of Castelfranco were evaluated. The bioactivity of polyphenol-rich extracts from chicories was studied in* in vitro* Caco-2 cell monolayers model. Morphological characteristics changes to test the antioxidant and/or prooxidant effect were verified by histological analysis and observed by Electronic Scansion Microscopy (SEM). On Caco-2 cell model, the polyphenols fractions from chicories have indicated a moderate antioxidant behavior until 17 *μ*M concentration, while 70 *μ*M and 34 *μ*M exert cytotoxic effects for Treviso's and Castelfranco's Chicory, respectively, highlighted by TEER decreasing, increased permeability, and alteration of epithelium. Our findings support the beneficial effects of these products in counteracting the oxidative stress and cellular damage, induced* in vitro* on Caco-2 cell model, through interaction with the mucopolysaccharide complexes in the glycocalyx, maintaining* in vivo* a healthy and effective intestinal barrier.

## 1. Introduction

Chicory, a plant genus typical of Mediterranean area, is native to Europe, Western Asia, and North America and its colour varies from white to red [1]. “Red Chicory of Treviso” and “Variegated Chicory of Castelfranco,” with PGI according to EU rules, are strongly linked to their territory and grown according to traditional cultivation techniques. These products have acquired great interest for their organoleptic and nutritional characteristics. As well known, the consumption of phytochemicals from fruits and vegetables can improve the prevention of several chronic degenerative pathologies [2, 3]. Phytochemicals content may be affected by several factors: genetic characteristic, environmental aspects, agronomic practices, and postharvest conditions [4, 5]. Genetic factors exert great influence on nutritional and phytochemicals content, between and within vegetables species. Climate condition, light exposure, temperature, relative humidity, and luminous intensity are specific parameters that affect food quality. In particular, the choice of an appropriate agronomic practice could improve the levels and the profile of bioactive compounds. Among the vegetal crops, red chicories are attractive because they may be consumed either raw or cooked. In particular, they are commonly eaten raw in salad during winter months when most vegetables are not in season. In fact, red chicories are particularly resistant to low temperatures [6] and their availability throughout the year is an important source of micronutrients during the coldest season. The red color is caused in large part by the presence of water-soluble pigments, anthocyanins, but several works show that the red-leaved varieties of* Cichorium intybus* L. have the highest content of polyphenols among the leafy vegetables that are consumed raw [7, 8]. Changes in phytochemicals content in agricultural production take on a particular importance in our diet. The bioactive compounds in foods, such as vitamins, carotenoids, and polyphenols, seem to be able to modulate one or more metabolic processes, which result in the promotion of better health [9]. The most accepted explanation for the protective effect of food probably derives from the observation that different plant phytochemicals may act as “scavengers” of free radicals, “quenchers” singlet oxygen, or metal chelators [10, 11] and therefore induce protection against oxidative damage through antibacterial, anti-inflammatory, hepatoprotective, anticarcinogenic, and vasodilator actions.

Recently, D'Evoli et al. [12] have shown that the high levels of antioxidant anthocyanins present in Red Chicory exert a direct scavenging effect against ROS formation in terms of antioxidant and cytoprotective activities as well as antiproliferative activity in Caco-2 cell. In the present work, we aimed to study the relationship between potential activity of polyphenol-rich extracts from chicories and morphological and chemical/physical changes in Caco-2 cellular line. To this purpose, bioactive compounds content (carotenoids and flavonoids) and total antioxidant activity were evaluated in Early and Late Red Chicory of Treviso and Variegated Chicory of Castelfranco. In addition, the bioactivity of polyphenol-rich extracts from chicories in* in vitro* Caco-2 cell monolayer model was studied. Morphological characteristics changes to test the antioxidant and/or prooxidant effect were verified by histological analysis and observed by Electronic Scansion Microscopy (SEM).

## 2. Materials and Methods

### 2.1. Reagents

All solvents were purchased from Carlo Erba (Milan, Italy), BDH (Poole, England), and Merck (Darmstadt, Germany). 2,4,6-Tri(2-pyridyl)-s-triazine (TPTZ) was from Fluka (Switzerland). Phosphate-buffered saline (PBS), 6-hydroxy-2,5,7,8-tetramethylchroman-2-carboxylic acid (Trolox), and ascorbic acid were provided by Sigma-Aldrich Srl. Commercial standards were also from Sigma-Aldrich Srl (Milan, Italy). AAPH (2,2′,-azobis (2-amidinopropane) dihydrochloride) (WACO Chem., Richmond, VA, USA) was used as a source of hydrophilic radicals. Double distilled water (Millipore, Milan, Italy) was used throughout the study.

### 2.2. *Cichorium intybus* L

Red Chicories of Treviso (two varieties: Early and Late) samples were grown in the region including Quinto (Treviso), Zero Branco (Treviso), and Scorzè (Venezia), while PGI samples of Variegated Chicory of Castelfranco come from Due Carrare (Padova), Mira (Venezia), and Monselice (Padova). A total weight of 5 kg of each variety for each locality was collected at harvesting time and delivered to the laboratory. Only the edible portion of the samples was utilized for analysis, according to the following methods.

### 2.3. Analytical Methods

The extraction of some flavonoids (quercetin, kaempferol, and apigenin) was performed as described by Hertog et al. [13] and the quantitative analysis through a system-ESA HPLC with electrochemical detector as reported by Azzini et al. [14]. Carotenoids were extracted from Treviso's and Castelfranco's chicory according to the method described by Sharpless et al. [15]. For the quantification, the samples were analyzed by high pressure liquid chromatography (HPLC) as reported by Maiani et al. [16]. Total Antioxidant Capacity (TAC) was evaluated using two different assays: Ferric Reducing Antioxidant Power (FRAP) [17] and Trolox Equivalent Antioxidant Capacity (TEAC) method [18, 19]. The results of each analysis have been performed in triplicate.

### 2.4. Transepithelial Electrical Resistance (TEER) Evaluation

For TEER evaluation, the polyphenol fractions from Red Chicory of Treviso and Variegated Chicory of Castelfranco hydrolysed extracts have been studied on Caco-2 cell monolayers to test the changes in tight-junction (TJ) permeability by TEER, the phenol red passage, and histological analysis.

The cellular line was treated with increasing concentration of two polyphenols extracts (0.2, 1.3, 10, 17, 34, and 70 *μ*M), for 180 minutes, to simulate* in vivo* physiological processes. The variations of transepithelial potentials and phenol red permeability were recorded at time intervals (30′). In the experiment, the cells were seeded onto polycarbonate filter cell culture chamber inserts (diameter 6.5 mm, area 0.33 cm^2^, and pore diameter 0.4 *μ*m), at density of 1.5 · 10^5^ cells per filter and placed in a multiwell Falcon. The filter divided the chamber into two parts, apical and basal, that represent the lumen and the basal area of the gastroenteric system. Into two chambers, TEER measurement for assessment of tight-junction permeability was performed using the Millicell ERS apparatus (Millipore, Bedford, MA, USA) according to the manufacturer's instruction. After calibrating the resistance system, the electrical resistance of the monolayer was measured by placing one electrode on either side of the polycarbonate filter [20, 21]. The results of each analysis have been performed in triplicate and expressed as OHM × cm^2^.

Cells reached confluence and differentiation within 15–20 days. During this time, cellular morphology was monitored and checked with a Leitz Diavert inverted light microscope.

### 2.5. Histological Analysis

**Figure 1:**
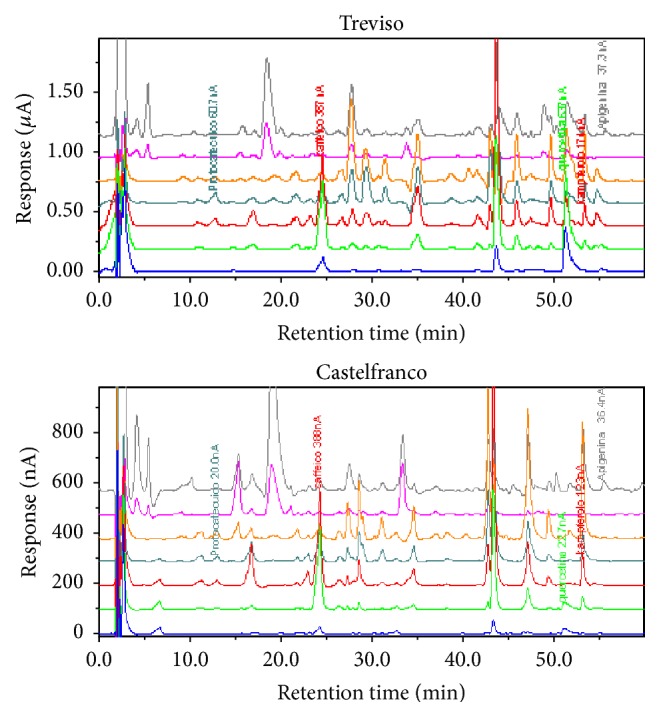
Representative HPLC-ECD gradient array chromatograms of polyphenol-rich extracts analyzed under specified conditions from Red Chicory of Treviso and Variegated of Castelfranco extracts.

The cellular monolayer was isolated together with the filter and fixed in Bouin (an aqueous solution composed of picric acid, acetic acid, and formaldehyde) for about 12 hours, then dehydrated using the alcohol ascending scale (70%, 80%, 90%, 95%, and 100%), and finally enclosed into resin blocks of polymerized epoxy (GMA, J134K polyscience Inc., Warrington, PA, USA). Then, the samples were glued on embedded stubs and cut into 3 *μ*m sections with a Micron rotary microtome (Zeiss Germany) appropriately assembled so as to use a crystal blade according to Ralph's modification [22, 23]. The sections glued to the microscope slides were stained with Harris' hematoxylin and eosin [24, 25]. The preparations were made permanent with slide covers and sealed with resin (Eukitt, mounting medium, BDA Laboratories Supplies Pool, England). All sections (3 *μ*m) were examined for histological changes by Diaplan 22 light microscopy (Leitz Germany) as shown in Figure 1. All pictures (microphotographies) have a magnification of 356x and were performed with Leica microscope Dialux 22.

### 2.6. SEM Analysis

At the end of each experiment (180 minutes), the cellular monolayer was fixed for 12 hours in formaldehyde (10%) and glutaraldehyde (2.5%) and then dried with alcohol solution. Then, the sample was dehydrated by Critical Point Drying (Emitech K850, Quorum Technologies, West Sussex, UK). The final dehydration was achieved in CO_2_. Finally, the sample was sputtered with gold for 120 s at 30 mA in a modified atmosphere with 2% Argon and analyzed using SEM (EVO LS10, Carl Zeiss Microscopy GmbH, Jena, Germany).

### 2.7. Statistics

The results are expressed as mean ± SD. Statistical data analysis was performed using the ANOVA test followed by Bonferroni post hoc test (significance at *P* < 0.05).

## 3. Results

### 3.1. Phytochemical Characterization

According to several authors, flavonoid components could be used as potential markers for the analysis of herbs and plants for human consumption [26–28].

With regard to the quantitative analysis of flavonoids (free plus conjugated forms), Figure 1 displays representative HPLC-DAD gradient array chromatograms, while their identification is reported in Table 1. The varied flavonoids content amongst different varieties and growing locations is summarized in Table 2. Comparing the growing locations, there were no significant differences in the flavonoids content, while significant differences were recorded between varieties. Quercetin was found as the most abundant flavonoid in Red Radish of Treviso varieties (90.04 ± 25.16 mg/kg and 97.88 ± 33.94 mg/kg, resp., in Late and Early Red Chicory) with respect to the Variegated Chicory of Castelfranco (14.20 ± 4.51 mg/kg).

The kaempferol was significantly different among cultivars and varieties. Its content in the Late Red Chicory of Treviso (22.80 ± 5.84 mg/kg) variety was significantly higher (*P* < 0.05) comparing to Early Red Chicory of Treviso (12.35 ± 7.65 mg/kg) and Variegated Chicory of Castelfranco (11.80 ± 3.64 mg/kg).

There were no significant differences in the apigenin content between cultivar and production area on average range from 2.60 ± 1.40 to 3.58 ± 0.29 mg/ kg, respectively, for Early Red Chicory of Treviso and Variegated Chicory of Castelfranco.

Our data (Table 3) indicate that lutein and *β*-carotene were the main carotenoids (ranging from 1.19 ± 0.24 to 2.40 ± 0.61 mg/kg and from 0.19 ± 0.02 to 0.48 ± 0.15 mg/kg, resp.). A significant difference (*P* < 0.05) was present between *β*-carotene content of Late Red Chicory of Treviso (0.38 ± 0.08 mg/kg) and Early Red Chicory of Treviso (0.22 ± 0.04 mg/kg). Variegated Chicory of Castelfranco showed a mean of *β*-carotene content equal to 0.35 ± 0.14 mg/kg. In the Early Red Chicory of Treviso, the mean lutein content was 2.16 ± 0.28 mg/kg with significantly higher levels (*P* < 0.05) than Late Red Chicory of Treviso (1.27 ± 0.20 mg/100 g) and Variegated Chicory of Castelfranco (1.20 ± 0.27 mg/100 g).

**Figure 2:**
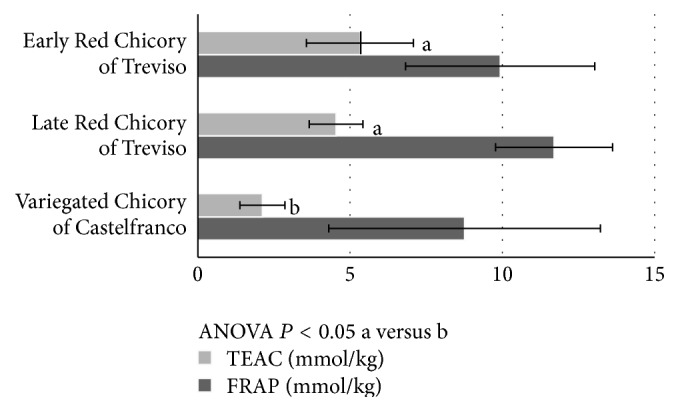
Total antioxidant capacity: FRAP (mmol/kg) and TEAC (mmol trolox/kg) values by different cultivars of* Cichorium intybus* L.

After evaluating the content of individual antioxidants, the cooperative action of bioactive molecules in the different specimens of* Cichorium intybus* L. was evaluated by total antioxidant capacity (TAC).  Figure 2 shows the synergistic effects of various antioxidants measured by FRAP (mmol/kg) and TEAC (mmol trolox/kg) methods.

Our results highlighted the highest FRAP values of Late Red and Early Red Chicories of Treviso with respect to Variegated Chicory of Castelfranco (11.70 ± 1.92 mmol/kg and 9.93 ± 3.11 mmol/kg versus 8.76 ± 4.46 mmol/kg, resp.). No differences by varieties and production area were present in FRAP (mmol trolox/kg) results.

Significant differences were present in TEAC (mmol trolox/kg) levels. TEAC values of Late Red Chicory of Treviso (4.54 ± 0.88 mmol trolox/kg) and Early Red Chicory of Treviso (5.32 ± 1.76 mmol trolox/kg) were higher (*P* < 0.05) compared with Variegated Chicory of Castelfranco (2.12 ± 0.74 mmol trolox/kg).

### 3.2. Cell-Based Assays

Differently from other food components, phytochemicals can exert their bioactivity without reaching the systemic circulation. Scarcely absorbed antioxidants might reach the large bowel contributing to protection from oxidative damage-induced gastrointestinal diseases [29]. There are several reports about pharmacological actions and anti-inflammatory effects of chicory [30, 31].

TEER measurements are routinely used to characterize monolayer integrity in the context of cell monolayer permeability experiments, or to quantify permeability changes, for example, as a consequence of paracellular permeability enhancers [32, 33].

**Figure 3:**
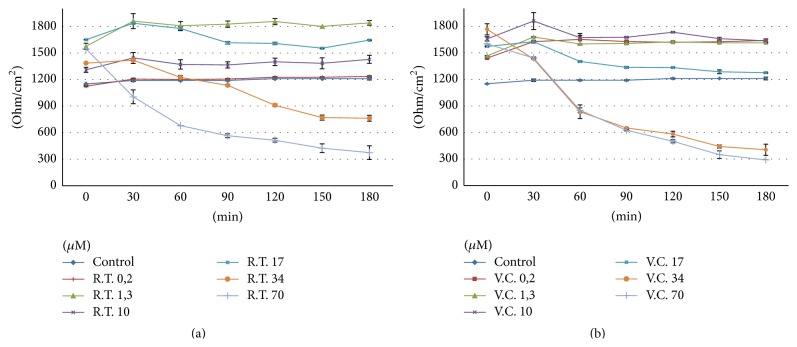
TEER changes at different concentration of polyphenol extract: (a) Red Chicory of Treviso (RT) and (b) Variegated Chicory of Castelfranco (VC) polyphenol extract.

Utilizing polyphenol fractions from Red Treviso's and Castelfranco's Chicory, the changes in TJs permeability were tested on Caco-2 cell line in monolayers culture. TEER measurements at increasing phenolic concentrations are shown in Figures 3(a) and 3(b), respectively, for Treviso Red Chicory and Variegated Chicory of Castelfranco.

**Figure 4:**
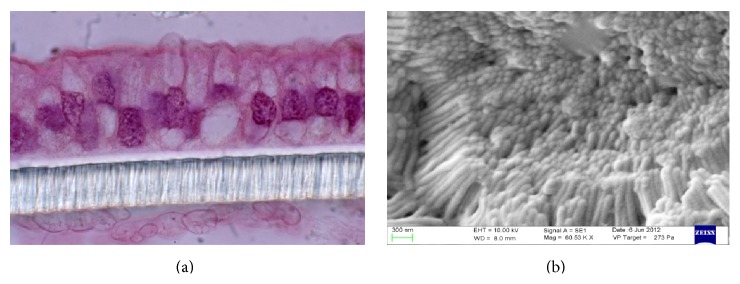
Histological analysis (a) and scanning electron micrographs (b) of normal Caco-2* pattern* treated with polyphenolics extract of Treviso Red (17 *μ*M).

**Figure 5:**
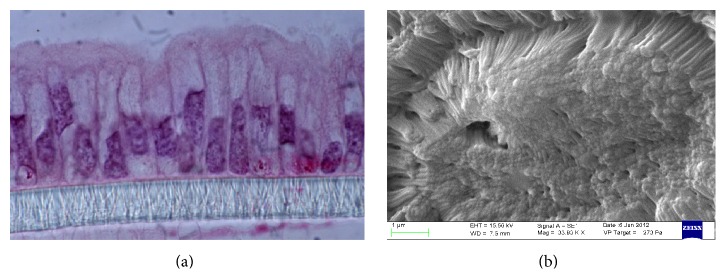
Histological analysis (a) and scanning electron micrographs (b) of normal Caco-2* pattern* treated with polyphenolics extract of Variegated Chicory of Castelfranco (17 *μ*M).

Upon treatment with Red Chicory of Treviso's and Variegated Chicory of Castelfranco's polyphenolics extract, as indicated by TEER values, the results showed a monolayer equilibrium model (healthy monolayer cells) at 0.2-1.3-10-17 *μ*M extract concentration. Until 17 *μ*M polyphenolic extracts concentration, the TEER measurements hold out a plateau, indicating the tightness of TJs and the absence of direct interaction between epithelial Caco-2 cells and chicory extracts as confirmed by histological analyses. Figures 4(a) and 4(b) display the 17 *μ*M effect upon both treatments. The ultrastructural cytological analysis by SEM highlighted and confirmed the absence of morphological change or extracts activity on the cellular monolayer (Figures 5(a) and 5(b)).

**Figure 6:**
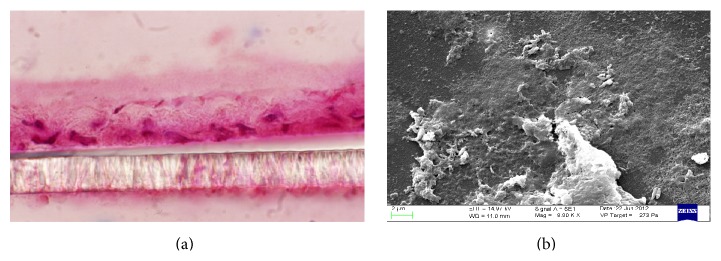
Histological analysis (a) and scanning electron micrographs (b) showing Caco-2 necrotic* pattern* upon treatment with polyphenolics extract: Treviso Red extract (70 *μ*M).

**Figure 7:**
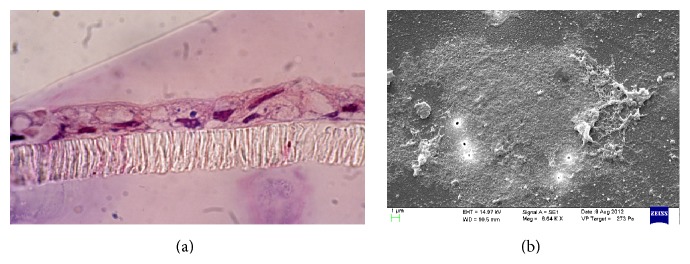
Histological analysis (a) and scanning electron micrographs (b) showing Caco-2 necrotic* pattern* upon treatment with polyphenolics extract: Variegated Chicory of Castelfranco extract (34 *μ*M).

The concentrations of 70 *μ*M and 34 *μ*M showed a high toxicity, respectively, for Treviso's and Castelfranco's Chicory extracts tested. These treatments produce lowering of TEER values (Figures 3(a) and 3(b)), highlighted by increased permeability of TJs (phenol red) and by alteration of epithelium. These concentrations promote cellular necrosis in Caco-2 cells monolayer as shown in histological analysis and confirmed by SEM observations (Figures 6(a), 6(b) and 7(a), 7(b) for Treviso's and Castelfranco's Chicory, resp.).

**Figure 8:**
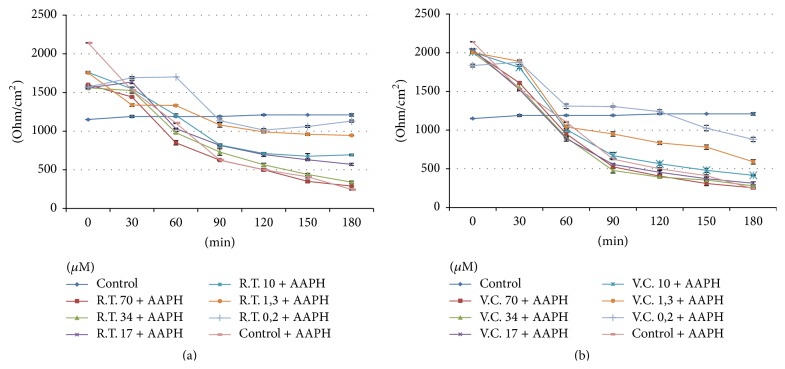
TEER changes at different concentration of polyphenol-rich extract after AAPH-induced oxidative stress. (a) Red Chicory of Treviso (RT) extract and (b) Variegated Chicory of Castelfranco (VC) polyphenol extract.

To better understand their bioactivity and attempting to demonstrate the probable prebiotic role of these extracts, Caco-2 oxidative stress was induced by adding 2,2′azobis (2-amidinopropane) dihydrochloride (AAPH) to the cell culture medium. The interactions between polyphenol-rich Red Chicory of Treviso extracts against AAPH-induced oxidative stress are reported in Figure 8(a). The progressive TEER increases indicated that, upon 0.2, 1.3, and 10 *μ*M, chicory extracts treatment displayed a strong antioxidant activity that appeared to be able to counteract the peroxidative trigger induced by AAPH, suggesting a cellular membrane integrity and confluency restoring. Only 0.2 *μ*M Variegated Chicory of Castelfranco extract exhibited a low antioxidant effect (Figure 8(b)) that promotes a cell damage recovery resulting by slight TEER increase.

## 4. Discussion

The antioxidant properties of several varieties of* Cichorium* genus vegetables have been attributed, in part, to the presence of phytochemicals including hydroxycinnamic acid derivatives, mono- and diglycosides of flavonoids, and anthocyanins. As reported by Rossetto et al. [6], the presence of these phenolics confers to red chicories an exceptionally high peroxyl radical scavenging activity in terms of both capacity and efficiency, particularly in their early stage of growth. The lower carotenoid values observed in the present study could be due to the limited exposure to sunlight and the lower temperature, during winter, because carotenoids biosynthesis is not stimulated as in vegetables in open fields. As reported by Niizu and Rodriguez-Amaya [34], the green chicory showed a higher average content of 53.7 ± 8.3 (mg/kg) and 35.3 ± 5.0 (mg/kg) for lutein and *β*-carotene, respectively. As discussed elsewhere [35], green chicory from Lazio exhibited higher carotenoids content. In particular, lutein and *β*-carotene values were 14.10 ± 3.30 mg/kg and 42.12 ± 5.99 mg/kg, respectively, in wild type, while the average content was 15.51 ± 3.28 mg/kg for lutein and 40.62 ± 2.15 mg/kg for *β*-carotene in cultivated chicory.

The total antioxidant capacity is a parameter strongly influenced by type of food (i.e., species and varieties for the same species), but also by growing conditions, environmental factors, and preservation techniques. Variegated Chicory of Castelfranco exhibited the lowest TAC values for the FRAP and TEAC methods, respectively. Red Chicory of Treviso (Early and Late) results, analyzed by the FRAP and TEAC assays, were according to Pellegrini et al. [36]. As reported elsewhere [35], changes in antioxidant composition of several vegetables were linked to agricultural practices and environmental factors. Regarding cell-based assays, TEER measurement represents a physical measure to evaluate the physiological state of cell cultures detecting the early intestinal barrier function* in vivo* damage. As known, defect in epithelial permeability caused by alteration of TJs is seen in several inflammatory bowel diseases. Different elements, including robust innate immune responses, epithelial paracellular permeability, and epithelial cell integrity, as well as the production of mucus, contribute to the integrity of the intestinal barrier [37]. Our experimental system was the confluent monolayer of Caco-2 cells at equilibrium. In this condition, the epithelial culture is more susceptible to variations of the chicory extracts concentration. So, small relative changes in concentrations of extracts correspond to chemical and physical altered cellular patterns and dramatic cell morphological changes. The tightness of TJs, as confirmed by histological analyses (Figures 4(a) and 5(a)) and the absence of morphological change or extracts activity on the cellular monolayer by SEM (Figures 4(b) and 5(b)), does not specify if these extracts maintain the protective effect of the antioxidant mixture but indicates the absence of direct interaction between epithelial Caco-2 cells and chicory extracts. These extracts could be compartmentalized in the mucopolysaccharides of the glycocalyx like prebiotic mixture. As known, the glycocalyx is a meshwork of glycoprotein molecules that binds to mucus largely composed of mucopolysaccharides produced by goblet cells. The glycocalyx and mucus form a flexible coat which provides cells protection from mechanical and chemical damage. Nutrients can diffuse into the mucosa, be acted upon by the enzymes in the glycocalyx, and create an area at high concentration of more easily adsorbed molecules by concentration gradient [38]. At the moment, our research is focused on clustering of the food extracts by glycocalyx interaction.

The greatest protective effect on cell cytotoxicity, deriving from Treviso Red radish extract (70 *μ*M) with respect to Variegated Chicory of Castelfranco extract (34 *μ*M), could be due to specific composition in polyphenol-rich fractions. The main flavonoids in above-mentioned extracts are reported in Table 1 and the TJs respond to various naturally plant-derived and food extracts. TEER measurements present dose-response curve patterns, indicating the absence of alteration in Caco-2 cell monolayer, as underlined by our results from 0.2 to 17 *μ*M chicory extracts treatments (Figure 3). Moreover,* Cichorium intybus* L. seems to counteract and improve the oxidative stress and cellular damage induced by AAPH* in vitro* Caco-2 cell model (Figure 8). As previously reported [39], when the prooxidant (AAPH) is added, the TEER measurements dramatically decrease until cellular necrosis; in addition, as reported by Finotti et al. [40], the presence of oxidant (AAPH) induces an increase in the mucopolysaccharides secretion located at microvilli glycocalyx. Overall, a better healthy action by Red Chicory of Treviso polyphenol-rich extract on Caco-2 (at 0.2-1.3-10 *μ*M concentrations) could be assumed which does not interact with monolayer cells while as exogenous substance it helps to maintain optimal cellular functions by its strong antioxidant activity. On the other hand, Red Chicory is characterized by a high content of anthocyanin pigments [41] that could exert several beneficial health or nutraceutical effects [12, 42, 43].

## 5. Conclusion

It could be concluded that the TJs response depends on the dose exposure and particular chemical composition of food extracts by synergic and interdependent antioxidant effects (enzymatic and nonenzymatic) with the epithelial glycocalyx. Even if the redox balance does not originate from a single cause, our study suggests that the interaction between antioxidant extracts and the mucopolysaccharide complexes in the glycocalyx could protect the* in vivo* lining of gut from damage maintaining a healthy and effective intestinal barrier.

## Figures and Tables

**Table 1:** Flavonoid identification from Red Chicory of Treviso and Variegated Chicory of Castelfranco extracts.

Peak analyte	Red Chicory of Treviso			Variegated Chicory of Castelfranco		
	RT (min)	Dominant channel height	Ratio accuracy	RT (min)	Dominant channel height	Ratio accuracy
Quercetin	51.26	637 nA	0.925	51.19	22.7 nA	0.947
Kaempferol	53.28	174 nA	0.800	53.13	123 nA	0.807
Apigenin	55.61	37.3 nA		55.52	36.4 nA	

**Table 2:** Flavonoids content amongst different varieties by growing locations (mg/kg).

	Quercetin (mg/kg)	Kaempferol (mg/kg)	Apigenin (mg/kg)
*Late Red Chicory of Treviso*			
Zero Branco	83.92 ± 15.08	21.43 ± 2.19	2.10 ± 0.61
Scorzè	95.89 ± 26.51	26.23 ± 10.48	3.24 ± 0.82
Quinto	90.31 ± 33.9	20.94 ± 4.86	3.05 ± 1.12

	90.04 ± 25.16^a^	22.80 ± 5.84^a^	2.80 ± 0.85

*Early Red Chicory of Treviso*			
Zero Branco	101.50 ± 40.20	14.42 ± 8.40	3.98 ± 1.81
Scorzè	100.23 ± 42.4	14.51 ± 9.80	1.51 ± 0.80
Quinto	91.89 ± 29.22	8.11 ± 5.38	2.30 ± 1.60

	97.88 ± 33.94^a^	12.35 ± 7.65^b^	2.60 ± 1.40

*Variegated Chicory of Castelfranco*			
Monselice	15.32 ± 3.54	8.68 ± 2.16	3.33 ± 0.44
Mira	14.47 ± 4.92	13.5 ± 3.81	3.78 ± 0.38
Due Carrare	12.8 ± 5.08	13.21 ± 4.97	3.62 ± 0.05

	14.20 ± 4.51^b^	11.80 ± 3.64^b^	3.58 ± 0.29

ANOVA: *P* < 0.05 a versus b by column.

**Table 3:** Carotenoids (lutein and *β*-carotene) means values (mg/kg) by different cultivars of *Chicorium intybus* L. and by production area.

	Lutein (mg/kg)	*beta*-carotene (mg/kg)
*Late Red Chicory of Treviso*		
Zero Branco	1.27 ± 0.17	0.27 ± 0.06
Scorzè	1.19 ± 0.24	0.20 ± 0.01
Quinto	1.41 ± 0.07	0.19 ± 0.02

	1.27 ± 0.20^b^	0.22 ± 0.04^b^

*Early Red Chicory of Treviso*		
Zero Branco	2.12 ± 0.89	0.45 ± 0.02
Scorzè	1.97 ± 0.11	0.35 ± 0.01
Quinto	2.40 ± 0.61	0.34 ± 0.09

	2.16 ± 0.28^a^	0.38 ± 0.08^a^

*Variegated Chicory of Castelfranco*		
Monselice	1.28 ± 0.13	0.48 ± 0.15
Mira	1.26 ± 0.36	0.35 ± 0.11
Due Carrare	1.07 ± 0.4	0.23 ± 0.01

	1.20 ± 0.27^b^	0.35 ± 0.14^ab^

ANOVA: *P* < 0.05 a versus b by column.

## Abbreviations

Caco-2: Human colon carcinoma cell line
FRAP: Ferric Reducing Antioxidant Power
TEAC: Trolox Equivalent Antioxidant Capacity
TEER: Transepithelial Electrical Resistance
TJ: Tight-junction
SEM: Scanning Electron Microscopy.
